# l-Tryptophan 4-nitro­phenol tris­olvate

**DOI:** 10.1107/S1600536812007817

**Published:** 2012-02-29

**Authors:** V. H. Rodrigues, M. M. R. R. Costa, M. Belsley, E. de Matos Gomes

**Affiliations:** a CEMDRX, Department of Physics, University of Coimbra, P-3004-516 Coimbra, Portugal; b Departamento de Física, Universidade do Minho, P-4710-057 Braga, Portugal

## Abstract

The title compound, C_11_H_12_N_2_O_2_·3C_6_H_5_NO_3_, comprises a zwitterionic amino acid formed by two nearly planar groups: (i) the indole ring and Cβ, and (ii) the carboxyl group, Cα, as well as the amine N atom, with r.m.s. deviations of 0.0084 and 0.0038 Å, respectively. The angle between these idealized planes is 39.47 (9)°. The amine group of the amino acid is in a *syn* (−*sc*) arrangement relative to the ring system. The overall crystal structure results from the packing of sheets parallel to the (001) planes. These sheets are formed by a pair of screw axis related parallel networks bound by hydrogen-bond and π–π stacking interactions. The intermolecular cohesion of all organic residues in each of the latter two-dimensional networks is achieved *via* strong hydrogen bonding, nitro–π and π–π stacking interactions.

## Related literature

For a general review on nonlinear optical properties of organic mol­ecules and crystals, see: Chemla & Zyss (1987 ▶); Zyss & Ledoux (1994 ▶); Zyss & Nicoud (1996 ▶). For similar and most common conformations of l-tryptophan, see: Bye *et al.* (1973 ▶); Bakke & Mostad (1980 ▶). For the Cambridge Structural Database, see: Allen (2002 ▶). For information on optical second harmonic generation (SHG) measurements, see: Kurtz & Perry (1968 ▶).
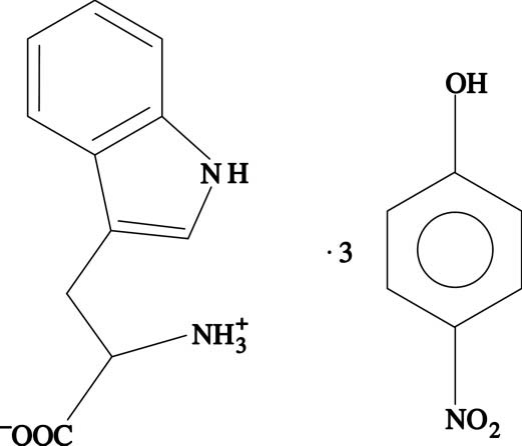


## Experimental

### 

#### Crystal data

C_11_H_12_N_2_O_2_·3C_6_H_5_NO_3_
*M* *_r_* = 621.56
Monoclinic, 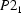

*a* = 13.0321 (9) Å
*b* = 6.7332 (4) Å
*c* = 17.3091 (10) Å
β = 104.479 (3)°
*V* = 1470.59 (16) Å^3^
*Z* = 2
Mo *K*α radiation
μ = 0.11 mm^−1^
*T* = 291 K
0.3 × 0.2 × 0.15 mm

#### Data collection

Bruker–Nonius APEXII CCD area-detector diffractometer
Absorption correction: multi-scan (*SADABS*; Sheldrick, 2003 ▶) *T*_min_ = 0.915, *T*_max_ = 0.980
94463 measured reflections
4289 independent reflections
2984 reflections with *I* > 2σ(*I*)
*R*_int_ = 0.031

#### Refinement

*R*[*F* ^2^ > 2σ(*F* ^2^)] = 0.035
*wR*(*F* ^2^) = 0.101
*S* = 1.04
4289 reflections
410 parameters
1 restraint
H-atom parameters constrained
Δρ_max_ = 0.14 e Å^−3^
Δρ_min_ = −0.15 e Å^−3^

### 

Data collection: *APEX2* (Bruker, 2003 ▶); cell refinement: *SAINT* (Bruker, 2003 ▶); data reduction: *SAINT*; program(s) used to solve structure: *SHELXS97* (Sheldrick, 2008 ▶); program(s) used to refine structure: *SHELXL97* (Sheldrick, 2008 ▶); molecular graphics: *PLATON* (Spek, 2009 ▶); software used to prepare material for publication: *SHELXL97*.

## Supplementary Material

Crystal structure: contains datablock(s) I, global. DOI: 10.1107/S1600536812007817/fk2052sup1.cif

Structure factors: contains datablock(s) I. DOI: 10.1107/S1600536812007817/fk2052Isup2.hkl

Supplementary material file. DOI: 10.1107/S1600536812007817/fk2052Isup3.cml

Additional supplementary materials:  crystallographic information; 3D view; checkCIF report

## Figures and Tables

**Table 1:** Hydrogen-bond geometry (Å, °)

*D*—H⋯*A*	*D*—H	H⋯*A*	*D*⋯*A*	*D*—H⋯*A*
N1—H1*B*⋯O21	0.89	2.10	2.970 (2)	165
N1—H1*C*⋯O1^i^	0.89	1.86	2.743 (2)	172
O43—H43*A*⋯O2^ii^	0.82	1.81	2.623 (2)	171
O23—H23*A*⋯O32^ii^	0.82	2.08	2.820 (3)	150
O33—H33*A*⋯O2^iii^	0.82	1.88	2.687 (2)	170

Symmetry codes: (i) 
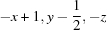
; (ii) 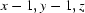; (iii) 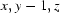.

**Fig. 1.**
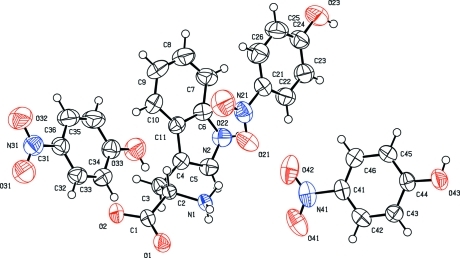
ORTEPII (Johnson, 1976) plot of the title compound. Displacement ellipsoids are drawn at the 50% level.

**Fig. 2.**
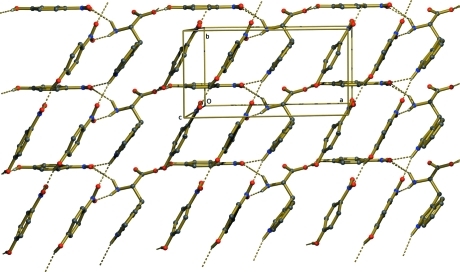
Representation of the two-dimensional networks, paralell to the (001) planes, of H-bonded molecules. Different sorts of chains can be individualized. Nitro-π and π-π stacking are also evident.

**Fig. 3.**
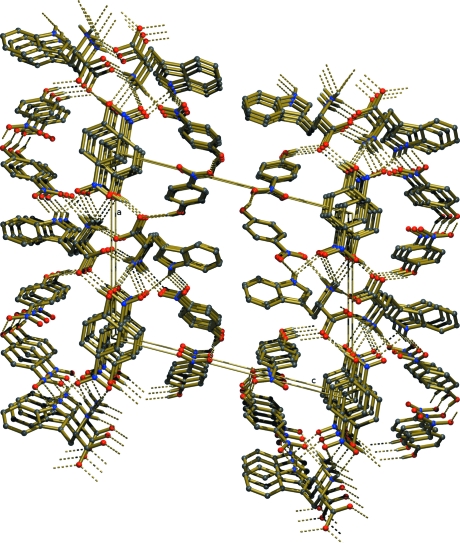
ac face view, showing the layered packing along the c axis of the two-dimensional networks parallel to (001) (shown in Fig. 2). H-bonded and π–π stacked pairs of screw axis related networks are visible.

## supplementary crystallographic information

### Comment

Non-linear optical phenomena (NLO) form the basis for a wide range of devices including frequency doublers, electro-optic modulators, optical limiters, high speed optical gates and parametric amplifiers. Organic molecules containing π electron systems asymetrized by donor and acceptor groups are highly polarizable entities which may give rise to organic polar crystals for NLO applications. The properties of individual molecules and their organization in the bulk crystalline structure are the key factors that determine the properties of the resulting molecular materials. An essential condition to obtain even-order NLO processes in materials is a noncentrosymmetric crystal structure. However, optimal molecular orientations are required if appreciable effects are to be achieved in molecular materials (Chemla & Zyss, 1987; Zyss & Ledoux, 1994; Zyss & Nicoud, 1996). In this context the crystal structure of the title compound resulting from co-crystallization of a chiral molecule, *L*-tryptophan, and *p*-nitrophenol, which has a high ground state dipole moment is reported. No other crystal structures with three neutral independent *p*-nitrophenol molecules were found in a search to the CSD database, version 1.13 (Allen, 2002).

The aminoacid is a zwitterion with overall neutral charge, which means that no Brønsted-Lowry acid-base reaction has occurred between *L*-tryptophan and *p*-nitrophenol molecules; this is further confirmed by inspection and comparison of all three C–O distances of the *p*-nitrophenol molecules which range from 1.341 (3) Å to 1.351 (3) Å.

The *L*-tryptophan has a conformation similar to the one found in the *L*-form of *DL*-formate (Bye *et al.*, 1973), with the amine group in a syn (-sc) arrangement relative to the indole and, as in most cases (Bakke & Mostad, 1980), with C1 *trans* to C4. The zwitterion can be divided in two planar groups forming an angle of 39.47 (9)°. The first group consists of the indole ring and C3; the second group includes the carboxyl group and the amine N atom. Root mean square deviations of the atoms in these latter planes are 0.0084 and 0.0038 Å, respectively.

Hydrogen bonded two-dimensional networks, parallel to the (001) planes, involving all four independent molecules, can be found in the crystal structure of the title compound. Figure 2 shows such a two-dimensional network of H-bonded, nitro-π and π-π stacked molecules. In the latter networks, chains formed by the aminoacid and one *p*-nitrophenol (X41—X46, where *X* stands for C, N or O) running along the *a* axis can be found. These chains are linked through two H-bonds: N1–H1A···O41*^i^* or N1–H1A···O42*^i^* and O43–H43A···O2*^iv^*. Other chains, formed by the same aminoacid and *p*-nitrophenol molecules, with a more mirregular shape, run along the *b* axis and are also connected through two H-bonds: the same bifurcated N1–H1A···O41*^i^* or N1–H1A···O42*^i^* and N2–H2A···O42. The other two *p*-nitrophenol molecules (X21—X26 and X31—X36, where *X* stands for C, N or O) can be seen as dimmers, H-bonded *via* O23–H23A···O32*^iv^*, that connect to the latter mdescribed base of perpendicularly running chains through H-bonds N1–H1B···O21, N2–H2A···O22*^iii^* and O33–H33···O2*^iii^*. All but one of the H-bonds found are used to build the two-dimensional network, which includes all the molecules present in the assymetric unit. Nitro-π and π-π interactions further stabilize the stacking of the latter dimmers and the indole ring along the *a* axis, *via* N31–O31···*Cg*_C21—C26_*^v^*, N21–O21···*Cg*_pyrrole_, N21–O21···*Cg*_ind. benz._ and correspondent π–π intermolecular contacts. Geometric details of the π-π and nitro-π interactions can be found in tables A and B. An H-bonded and also π-π stacked pair of the latter two-dimensional networks (screw axis related to each other), constitutes a sheet (parallel to the (001) planes); the relevant intermolecular contacts in the binding of this pair of neighbouring two-dimensional networks are the H-bond N1–H1C···O1^ii^ and π-π stacking interactions *Cg*_ind. benz._···*Cg*_C21—C26_^vi^ and *Cg*_C41—C46_···*Cg*_C41—C46_^vii^ (geometric details can be found in table A). The overall three-dimensional structure can be viewed as a repetition along the *c* axis of the latter sheets, as shown in Fig. 3.

Optical second harmonic generation (SHG) was measured on polycrystalline samples using the standard Kurtz and Perry technique (Kurtz and Perry, 1968). The material was particle sized using a set of standard microsieves (Retsch) having mesh width between 50 µm and 160µm. The sample cell consisted of a microscope slide with a depression of 3 mm diameter and 0.5 mm thickness covered with a flat microscope slide. The generated second harmonic signal was compared with that generated by polycrystalline urea with the same grain size and similar sample preparation. The *L*-tryptophan tris(4-nitrophenol) SHG response was twice that of urea.

Symmetry codes: (i) *x*, *y* + 1, 1 - *z*; (ii) -*x* + 1, *y* - 1/2, -*z*; (iii) *x* ,*y* - 1, *z*; (iv) *x* - 1, *y* - 1, *z*; (v) *x* + 1, *y*, *z*; (vi) -*x* + 1, *y* + 1/2, -*z* + 1; (vii) -*x*, *y* + 1/2, -*z*.

### Experimental

Single crystals were produced by dissolving 5.0 mmol of *L*-tryptophan and 10 mmol of *p*-nitrophenol in 30 ml of hot methanol. Yellow needles were formed by slow cooling at room temperature.

### Refinement

The structure was solved by direct methods using *SHELXS97* (Sheldrick, 2008). All H(C/N) atoms were placed at idealized positions and refined as riding [C—H=0.93 (aromatic C)— 0.98Å, *U*_iso_(H)=1.2*U*_eq_(C); N—H=0.89Å and *U*_iso_(H)=1.5*U*_eq_(N) (amino N), N—H=0.86Å and *U*_iso_(H)=1.2*U*_eq_(N) (aromatic N)]. Hydroxyl H atoms have been positioned and refined using HFIX 147 with *SHELXL* (Sheldrick, 2008).

Examination of the crystal structure with *PLATON* (Spek, 2009) showed that there are no solvent-accessible voids in the crystal lattice.

The refined model structure is non-centrosymmetric with only atoms which are poor anomalous scatterers for the wavelength used, therefore Friedel pairs were merged before the final refinement.

### Crystal data

**Table:**

C_11_H_12_N_2_O_2_·3C_6_H_5_NO_3_	*F*(000) = 648
*M**_r_* = 621.56	*D*_x_ = 1.404 Mg m^−^^3^
Monoclinic, *P*2_1_	Mo *K*α radiation, λ = 0.71073 Å
Hall symbol: P 2yb	Cell parameters from 8205 reflections
*a* = 13.0321 (9) Å	θ = 2.6–25.8°
*b* = 6.7332 (4) Å	µ = 0.11 mm^−^^1^
*c* = 17.3091 (10) Å	*T* = 291 K
β = 104.479 (3)°	Block, yellow
*V* = 1470.59 (16) Å^3^	0.3 × 0.2 × 0.15 mm
*Z* = 2	

### Data collection

**Table:**

Bruker–Nonius APEXII CCD area-detector diffractometer	4289 independent reflections
Radiation source: fine-focus sealed tube	2984 reflections with *I* > 2σ(*I*)
Graphite monochromator	*R*_int_ = 0.031
φ and ω scans	θ_max_ = 30.9°, θ_min_ = 3.2°
Absorption correction: multi-scan (*SADABS*; Sheldrick, 2003)	*h* = −18→17
*T*_min_ = 0.915, *T*_max_ = 0.980	*k* = −8→9
94463 measured reflections	*l* = −22→21

### Refinement

**Table:**

Refinement on *F*^2^	Primary atom site location: structure-invariant direct methods
Least-squares matrix: full	Secondary atom site location: difference Fourier map
*R*[*F*^2^ > 2σ(*F*^2^)] = 0.035	Hydrogen site location: inferred from neighbouring sites
*wR*(*F*^2^) = 0.101	H-atom parameters constrained
*S* = 1.04	*w* = 1/[σ^2^(*F*_o_^2^) + (0.0546*P*)^2^ + 0.0968*P*] where *P* = (*F*_o_^2^ + 2*F*_c_^2^)/3
4289 reflections	(Δ/σ)_max_ < 0.001
410 parameters	Δρ_max_ = 0.14 e Å^−^^3^
1 restraint	Δρ_min_ = −0.15 e Å^−^^3^

### Special details

**Table:**

Geometry. All e.s.d.'s (except the e.s.d. in the dihedral angle between two l.s. planes) are estimated using the full covariance matrix. The cell e.s.d.'s are taken into account individually in the estimation of e.s.d.'s in distances, angles and torsion angles; correlations between e.s.d.'s in cell parameters are only used when they are defined by crystal symmetry. An approximate (isotropic) treatment of cell e.s.d.'s is used for estimating e.s.d.'s involving l.s. planes.
Refinement. Refinement of *F*^2^ against ALL reflections. The weighted *R*-factor *wR* and goodness of fit *S* are based on *F*^2^, conventional *R*-factors *R* are based on *F*, with *F* set to zero for negative *F*^2^. The threshold expression of *F*^2^ > σ(*F*^2^) is used only for calculating *R*-factors(gt) *etc*. and is not relevant to the choice of reflections for refinement. *R*-factors based on *F*^2^ are statistically about twice as large as those based on *F*, and *R*- factors based on ALL data will be even larger.

### Fractional atomic coordinates and isotropic or equivalent isotropic displacement parameters (Å^2^)

**Table:**

	*x*	*y*	*z*	*U*_iso_*/*U*_eq_	
O1	0.53801 (10)	1.2043 (3)	0.02152 (8)	0.0593 (4)	
O2	0.68508 (10)	1.2042 (3)	0.12018 (8)	0.0595 (4)	
C1	0.59036 (14)	1.1548 (3)	0.08874 (11)	0.0431 (4)	
C2	0.53789 (12)	1.0190 (3)	0.13787 (10)	0.0387 (4)	
H2	0.5327	1.0894	0.1863	0.046*	
N1	0.42901 (10)	0.9709 (3)	0.08966 (9)	0.0411 (3)	
H1A	0.3969	1.0818	0.0683	0.062*	
H1B	0.3921	0.9159	0.1209	0.062*	
H1C	0.4330	0.8864	0.0510	0.062*	
C3	0.60036 (14)	0.8278 (3)	0.16154 (11)	0.0457 (4)	
H3A	0.6082	0.7614	0.1136	0.055*	
H3B	0.6707	0.8611	0.1933	0.055*	
C4	0.55002 (14)	0.6876 (3)	0.20785 (11)	0.0441 (4)	
C5	0.48396 (16)	0.5339 (3)	0.17877 (11)	0.0507 (5)	
H5	0.4631	0.4972	0.1253	0.061*	
N2	0.45239 (15)	0.4408 (3)	0.23941 (10)	0.0573 (4)	
H2A	0.4110	0.3396	0.2342	0.069*	
C6	0.49761 (16)	0.5353 (3)	0.30952 (11)	0.0511 (5)	
C7	0.48737 (19)	0.4966 (4)	0.38646 (13)	0.0653 (6)	
H7	0.4465	0.3918	0.3971	0.078*	
C8	0.5409 (2)	0.6214 (6)	0.44572 (14)	0.0815 (8)	
H8	0.5356	0.6007	0.4977	0.098*	
C9	0.6023 (2)	0.7764 (6)	0.43064 (13)	0.0840 (9)	
H9	0.6372	0.8571	0.4726	0.101*	
C10	0.61303 (18)	0.8147 (5)	0.35448 (13)	0.0666 (6)	
H10	0.6552	0.9187	0.3450	0.080*	
C11	0.55904 (15)	0.6934 (3)	0.29236 (11)	0.0470 (4)	
O41	0.24461 (14)	0.2491 (4)	−0.00160 (14)	0.0952 (7)	
O42	0.25999 (13)	0.2738 (4)	0.12387 (14)	0.0957 (6)	
N41	0.20582 (15)	0.2621 (3)	0.05568 (14)	0.0674 (5)	
C41	0.09109 (14)	0.2606 (3)	0.04219 (12)	0.0481 (4)	
C42	0.02939 (15)	0.2653 (3)	−0.03489 (12)	0.0526 (5)	
H42	0.0606	0.2673	−0.0777	0.063*	
C43	−0.07909 (15)	0.2669 (3)	−0.04782 (11)	0.0506 (5)	
H43	−0.1217	0.2729	−0.0996	0.061*	
C44	−0.12507 (14)	0.2595 (3)	0.01613 (11)	0.0472 (4)	
O43	−0.23142 (11)	0.2620 (3)	−0.00108 (10)	0.0700 (5)	
H43A	−0.2510	0.2426	0.0398	0.105*	
C45	−0.06176 (15)	0.2499 (3)	0.09340 (11)	0.0479 (4)	
H45	−0.0927	0.2422	0.1362	0.058*	
C46	0.04710 (15)	0.2517 (3)	0.10678 (12)	0.0509 (5)	
H46	0.0901	0.2469	0.1585	0.061*	
O21	0.29993 (15)	0.8641 (4)	0.20268 (11)	0.0833 (6)	
O22	0.3440 (2)	1.0653 (4)	0.30178 (14)	0.1070 (8)	
N21	0.30274 (16)	0.9127 (3)	0.27141 (13)	0.0665 (5)	
C21	0.25695 (16)	0.7792 (4)	0.31921 (12)	0.0538 (5)	
C22	0.21164 (19)	0.6059 (4)	0.28520 (14)	0.0625 (6)	
H22	0.2096	0.5775	0.2323	0.075*	
C23	0.1695 (2)	0.4750 (4)	0.32914 (14)	0.0686 (6)	
H23	0.1380	0.3582	0.3060	0.082*	
C24	0.17371 (18)	0.5165 (4)	0.40792 (13)	0.0644 (6)	
O23	0.13486 (17)	0.3904 (4)	0.45435 (11)	0.0935 (7)	
H23A	0.0916	0.3157	0.4260	0.140*	
C25	0.2203 (2)	0.6886 (5)	0.44203 (14)	0.0770 (8)	
H25	0.2238	0.7151	0.4954	0.092*	
C26	0.2620 (2)	0.8231 (4)	0.39763 (15)	0.0732 (7)	
H26	0.2929	0.9408	0.4204	0.088*	
O31	1.00715 (17)	1.0726 (4)	0.28193 (12)	0.0961 (7)	
O32	1.01880 (18)	1.0385 (4)	0.40723 (12)	0.0973 (7)	
N31	0.98754 (16)	0.9822 (4)	0.33756 (12)	0.0685 (5)	
C31	0.92836 (16)	0.7970 (4)	0.32165 (12)	0.0544 (5)	
C32	0.88446 (16)	0.7437 (4)	0.24380 (12)	0.0562 (5)	
H32	0.8918	0.8261	0.2024	0.067*	
C33	0.82972 (17)	0.5685 (4)	0.22744 (12)	0.0549 (5)	
H33	0.7998	0.5322	0.1748	0.066*	
C34	0.81888 (17)	0.4453 (4)	0.28894 (13)	0.0585 (5)	
O33	0.76777 (16)	0.2709 (3)	0.27645 (11)	0.0824 (5)	
H33A	0.7372	0.2606	0.2291	0.124*	
C35	0.8646 (2)	0.5020 (5)	0.36739 (14)	0.0821 (8)	
H35	0.8588	0.4191	0.4091	0.098*	
C36	0.9181 (2)	0.6787 (5)	0.38391 (14)	0.0752 (7)	
H36	0.9469	0.7177	0.4364	0.090*	

### Atomic displacement parameters (Å^2^)

**Table:**

	*U*^11^	*U*^22^	*U*^33^	*U*^12^	*U*^13^	*U*^23^
O1	0.0484 (7)	0.0692 (10)	0.0588 (8)	−0.0064 (7)	0.0106 (6)	0.0227 (8)
O2	0.0432 (7)	0.0694 (11)	0.0653 (8)	−0.0176 (7)	0.0121 (6)	−0.0004 (8)
C1	0.0386 (9)	0.0416 (10)	0.0515 (10)	−0.0029 (8)	0.0154 (8)	−0.0011 (8)
C2	0.0349 (8)	0.0410 (10)	0.0410 (8)	−0.0016 (7)	0.0108 (7)	−0.0020 (8)
N1	0.0333 (7)	0.0421 (8)	0.0505 (8)	0.0011 (6)	0.0153 (6)	0.0007 (7)
C3	0.0391 (9)	0.0464 (10)	0.0521 (10)	0.0047 (8)	0.0123 (8)	0.0044 (9)
C4	0.0443 (9)	0.0396 (10)	0.0478 (10)	0.0062 (8)	0.0104 (7)	0.0036 (8)
C5	0.0635 (12)	0.0403 (10)	0.0499 (10)	−0.0009 (10)	0.0171 (9)	−0.0035 (9)
N2	0.0741 (11)	0.0402 (9)	0.0593 (10)	−0.0111 (9)	0.0197 (8)	−0.0020 (8)
C6	0.0575 (11)	0.0447 (10)	0.0504 (10)	0.0055 (9)	0.0123 (8)	0.0055 (9)
C7	0.0746 (14)	0.0681 (16)	0.0557 (11)	0.0048 (13)	0.0212 (10)	0.0141 (12)
C8	0.0835 (17)	0.110 (2)	0.0482 (12)	0.0041 (18)	0.0105 (12)	0.0046 (15)
C9	0.0823 (16)	0.113 (3)	0.0483 (12)	−0.0096 (19)	0.0004 (11)	−0.0162 (16)
C10	0.0571 (12)	0.0776 (17)	0.0593 (12)	−0.0122 (12)	0.0037 (10)	−0.0101 (12)
C11	0.0442 (9)	0.0474 (11)	0.0468 (10)	0.0056 (8)	0.0066 (8)	0.0028 (9)
O41	0.0646 (10)	0.0947 (15)	0.1429 (18)	0.0118 (12)	0.0570 (11)	0.0057 (15)
O42	0.0514 (9)	0.0956 (16)	0.1276 (16)	0.0020 (11)	−0.0014 (10)	−0.0095 (15)
N41	0.0489 (10)	0.0436 (10)	0.1112 (16)	0.0049 (9)	0.0228 (11)	−0.0014 (12)
C41	0.0410 (9)	0.0340 (9)	0.0710 (12)	0.0021 (8)	0.0174 (8)	−0.0011 (10)
C42	0.0617 (11)	0.0385 (10)	0.0663 (12)	0.0026 (10)	0.0325 (10)	0.0004 (10)
C43	0.0547 (11)	0.0464 (11)	0.0499 (10)	−0.0009 (10)	0.0119 (8)	0.0053 (9)
C44	0.0424 (9)	0.0389 (9)	0.0611 (11)	0.0006 (9)	0.0147 (8)	0.0073 (9)
O43	0.0413 (7)	0.0937 (13)	0.0752 (10)	0.0014 (9)	0.0149 (6)	0.0230 (11)
C45	0.0536 (10)	0.0420 (10)	0.0525 (10)	0.0043 (9)	0.0213 (8)	0.0042 (9)
C46	0.0529 (10)	0.0405 (10)	0.0558 (10)	0.0044 (10)	0.0070 (8)	−0.0020 (9)
O21	0.0852 (12)	0.0988 (16)	0.0757 (11)	−0.0139 (11)	0.0384 (9)	0.0120 (11)
O22	0.1260 (18)	0.0763 (15)	0.1163 (16)	−0.0442 (14)	0.0257 (13)	0.0043 (13)
N21	0.0608 (11)	0.0620 (13)	0.0786 (14)	−0.0089 (10)	0.0208 (10)	0.0087 (11)
C21	0.0505 (10)	0.0533 (12)	0.0603 (11)	−0.0003 (10)	0.0189 (9)	0.0036 (11)
C22	0.0698 (14)	0.0650 (14)	0.0554 (11)	−0.0094 (12)	0.0208 (10)	−0.0022 (11)
C23	0.0774 (15)	0.0668 (15)	0.0621 (12)	−0.0181 (14)	0.0188 (11)	−0.0015 (12)
C24	0.0637 (13)	0.0717 (16)	0.0616 (12)	−0.0033 (13)	0.0226 (10)	0.0093 (13)
O23	0.1037 (15)	0.1068 (18)	0.0754 (11)	−0.0259 (13)	0.0328 (11)	0.0195 (12)
C25	0.0969 (18)	0.0840 (19)	0.0577 (13)	−0.0073 (16)	0.0337 (13)	−0.0110 (13)
C26	0.0859 (17)	0.0645 (15)	0.0702 (14)	−0.0093 (14)	0.0214 (12)	−0.0138 (13)
O31	0.1105 (15)	0.0881 (16)	0.0887 (13)	−0.0350 (13)	0.0233 (11)	0.0127 (12)
O32	0.1210 (16)	0.0899 (16)	0.0789 (11)	−0.0343 (14)	0.0210 (11)	−0.0231 (12)
N31	0.0688 (12)	0.0676 (13)	0.0687 (12)	−0.0046 (11)	0.0166 (9)	−0.0065 (11)
C31	0.0539 (11)	0.0559 (13)	0.0555 (11)	−0.0016 (10)	0.0176 (9)	−0.0041 (10)
C32	0.0600 (11)	0.0571 (13)	0.0515 (11)	0.0059 (11)	0.0141 (9)	0.0073 (10)
C33	0.0567 (12)	0.0565 (13)	0.0488 (10)	0.0055 (11)	0.0082 (8)	−0.0017 (10)
C34	0.0574 (12)	0.0571 (13)	0.0605 (12)	−0.0010 (11)	0.0135 (10)	0.0004 (11)
O33	0.0906 (12)	0.0718 (12)	0.0788 (10)	−0.0230 (11)	0.0102 (9)	0.0056 (10)
C35	0.108 (2)	0.086 (2)	0.0551 (13)	−0.0258 (18)	0.0252 (13)	0.0055 (14)
C36	0.0945 (18)	0.0815 (18)	0.0515 (12)	−0.0182 (16)	0.0219 (12)	−0.0084 (13)

### Geometric parameters (Å, º)

**Table:**

O1—C1	1.238 (2)	C44—O43	1.343 (2)
O2—C1	1.263 (2)	C44—C45	1.386 (3)
C1—C2	1.522 (2)	O43—H43A	0.8200
C2—N1	1.491 (2)	C45—C46	1.379 (3)
C2—C3	1.524 (3)	C45—H45	0.9300
C2—H2	0.9800	C46—H46	0.9300
N1—H1A	0.8900	O21—N21	1.225 (3)
N1—H1B	0.8900	O22—N21	1.217 (3)
N1—H1C	0.8900	N21—C21	1.448 (3)
C3—C4	1.492 (3)	C21—C22	1.372 (3)
C3—H3A	0.9700	C21—C26	1.375 (3)
C3—H3B	0.9700	C22—C23	1.366 (3)
C4—C5	1.360 (3)	C22—H22	0.9300
C4—C11	1.438 (3)	C23—C24	1.380 (3)
C5—N2	1.371 (3)	C23—H23	0.9300
C5—H5	0.9300	C24—O23	1.351 (3)
N2—C6	1.367 (3)	C24—C25	1.371 (4)
N2—H2A	0.8600	O23—H23A	0.8200
C6—C7	1.396 (3)	C25—C26	1.384 (4)
C6—C11	1.407 (3)	C25—H25	0.9300
C7—C8	1.374 (4)	C26—H26	0.9300
C7—H7	0.9300	O31—N31	1.219 (3)
C8—C9	1.379 (5)	O32—N31	1.232 (3)
C8—H8	0.9300	N31—C31	1.456 (3)
C9—C10	1.384 (3)	C31—C36	1.373 (3)
C9—H9	0.9300	C31—C32	1.373 (3)
C10—C11	1.393 (3)	C32—C33	1.371 (4)
C10—H10	0.9300	C32—H32	0.9300
O41—N41	1.223 (3)	C33—C34	1.384 (3)
O42—N41	1.217 (3)	C33—H33	0.9300
N41—C41	1.455 (3)	C34—O33	1.341 (3)
C41—C42	1.375 (3)	C34—C35	1.393 (3)
C41—C46	1.379 (3)	O33—H33A	0.8200
C42—C43	1.375 (3)	C35—C36	1.373 (4)
C42—H42	0.9300	C35—H35	0.9300
C43—C44	1.385 (3)	C36—H36	0.9300
C43—H43	0.9300		
			
O1—C1—O2	125.70 (17)	C42—C43—C44	120.16 (18)
O1—C1—C2	117.88 (15)	C42—C43—H43	119.9
O2—C1—C2	116.40 (16)	C44—C43—H43	119.9
N1—C2—C1	108.38 (14)	O43—C44—C43	116.81 (17)
N1—C2—C3	109.67 (15)	O43—C44—C45	123.14 (17)
C1—C2—C3	111.92 (13)	C43—C44—C45	120.04 (17)
N1—C2—H2	108.9	C44—O43—H43A	109.5
C1—C2—H2	108.9	C46—C45—C44	120.04 (17)
C3—C2—H2	108.9	C46—C45—H45	120.0
C2—N1—H1A	109.5	C44—C45—H45	120.0
C2—N1—H1B	109.5	C45—C46—C41	118.87 (17)
H1A—N1—H1B	109.5	C45—C46—H46	120.6
C2—N1—H1C	109.5	C41—C46—H46	120.6
H1A—N1—H1C	109.5	O22—N21—O21	123.2 (2)
H1B—N1—H1C	109.5	O22—N21—C21	118.6 (2)
C4—C3—C2	113.69 (14)	O21—N21—C21	118.2 (2)
C4—C3—H3A	108.8	C22—C21—C26	121.1 (2)
C2—C3—H3A	108.8	C22—C21—N21	118.5 (2)
C4—C3—H3B	108.8	C26—C21—N21	120.4 (2)
C2—C3—H3B	108.8	C23—C22—C21	120.0 (2)
H3A—C3—H3B	107.7	C23—C22—H22	120.0
C5—C4—C11	106.28 (17)	C21—C22—H22	120.0
C5—C4—C3	127.24 (17)	C22—C23—C24	119.9 (2)
C11—C4—C3	126.45 (18)	C22—C23—H23	120.1
C4—C5—N2	110.37 (17)	C24—C23—H23	120.1
C4—C5—H5	124.8	O23—C24—C25	117.8 (2)
N2—C5—H5	124.8	O23—C24—C23	122.2 (3)
C6—N2—C5	108.74 (17)	C25—C24—C23	120.0 (2)
C6—N2—H2A	125.6	C24—O23—H23A	109.5
C5—N2—H2A	125.6	C24—C25—C26	120.5 (2)
N2—C6—C7	129.6 (2)	C24—C25—H25	119.8
N2—C6—C11	107.86 (16)	C26—C25—H25	119.8
C7—C6—C11	122.5 (2)	C21—C26—C25	118.6 (2)
C8—C7—C6	116.5 (3)	C21—C26—H26	120.7
C8—C7—H7	121.8	C25—C26—H26	120.7
C6—C7—H7	121.8	O31—N31—O32	122.4 (2)
C7—C8—C9	122.1 (2)	O31—N31—C31	119.0 (2)
C7—C8—H8	118.9	O32—N31—C31	118.6 (2)
C9—C8—H8	118.9	C36—C31—C32	121.4 (2)
C8—C9—C10	121.5 (3)	C36—C31—N31	119.9 (2)
C8—C9—H9	119.2	C32—C31—N31	118.7 (2)
C10—C9—H9	119.2	C33—C32—C31	119.7 (2)
C9—C10—C11	118.3 (3)	C33—C32—H32	120.2
C9—C10—H10	120.8	C31—C32—H32	120.2
C11—C10—H10	120.8	C32—C33—C34	120.3 (2)
C10—C11—C6	118.99 (19)	C32—C33—H33	119.8
C10—C11—C4	134.3 (2)	C34—C33—H33	119.8
C6—C11—C4	106.74 (17)	O33—C34—C33	122.9 (2)
O42—N41—O41	122.2 (2)	O33—C34—C35	118.1 (2)
O42—N41—C41	118.7 (2)	C33—C34—C35	119.0 (2)
O41—N41—C41	119.1 (2)	C34—O33—H33A	109.5
C42—C41—C46	121.78 (17)	C36—C35—C34	120.8 (2)
C42—C41—N41	118.93 (19)	C36—C35—H35	119.6
C46—C41—N41	119.28 (19)	C34—C35—H35	119.6
C43—C42—C41	119.08 (18)	C35—C36—C31	118.9 (2)
C43—C42—H42	120.5	C35—C36—H36	120.6
C41—C42—H42	120.5	C31—C36—H36	120.6
			
O1—C1—C2—N1	1.2 (2)	C42—C43—C44—O43	179.9 (2)
O2—C1—C2—N1	−177.61 (17)	C42—C43—C44—C45	−0.2 (3)
O1—C1—C2—C3	122.22 (19)	O43—C44—C45—C46	−178.8 (2)
O2—C1—C2—C3	−56.5 (2)	C43—C44—C45—C46	1.3 (3)
N1—C2—C3—C4	−58.0 (2)	C44—C45—C46—C41	−0.8 (3)
C1—C2—C3—C4	−178.34 (15)	C42—C41—C46—C45	−0.8 (3)
C2—C3—C4—C5	94.3 (2)	N41—C41—C46—C45	−179.85 (19)
C2—C3—C4—C11	−83.3 (2)	O22—N21—C21—C22	−179.2 (2)
C11—C4—C5—N2	−1.0 (2)	O21—N21—C21—C22	−0.7 (3)
C3—C4—C5—N2	−179.01 (18)	O22—N21—C21—C26	−1.2 (4)
C4—C5—N2—C6	0.5 (2)	O21—N21—C21—C26	177.3 (2)
C5—N2—C6—C7	178.8 (2)	C26—C21—C22—C23	0.8 (4)
C5—N2—C6—C11	0.3 (2)	N21—C21—C22—C23	178.8 (2)
N2—C6—C7—C8	−178.4 (2)	C21—C22—C23—C24	−0.7 (4)
C11—C6—C7—C8	−0.1 (3)	C22—C23—C24—O23	−178.8 (3)
C6—C7—C8—C9	−0.4 (4)	C22—C23—C24—C25	−0.1 (4)
C7—C8—C9—C10	0.1 (5)	O23—C24—C25—C26	179.7 (3)
C8—C9—C10—C11	0.7 (4)	C23—C24—C25—C26	0.9 (4)
C9—C10—C11—C6	−1.2 (3)	C22—C21—C26—C25	−0.1 (4)
C9—C10—C11—C4	179.4 (2)	N21—C21—C26—C25	−178.0 (2)
N2—C6—C11—C10	179.5 (2)	C24—C25—C26—C21	−0.8 (4)
C7—C6—C11—C10	0.9 (3)	O31—N31—C31—C36	170.9 (3)
N2—C6—C11—C4	−0.9 (2)	O32—N31—C31—C36	−6.9 (3)
C7—C6—C11—C4	−179.5 (2)	O31—N31—C31—C32	−8.5 (3)
C5—C4—C11—C10	−179.3 (2)	O32—N31—C31—C32	173.7 (2)
C3—C4—C11—C10	−1.3 (4)	C36—C31—C32—C33	−0.4 (3)
C5—C4—C11—C6	1.2 (2)	N31—C31—C32—C33	179.0 (2)
C3—C4—C11—C6	179.19 (18)	C31—C32—C33—C34	−0.2 (3)
O42—N41—C41—C42	174.3 (2)	C32—C33—C34—O33	−178.9 (2)
O41—N41—C41—C42	−6.6 (3)	C32—C33—C34—C35	−0.2 (3)
O42—N41—C41—C46	−6.7 (3)	O33—C34—C35—C36	179.9 (3)
O41—N41—C41—C46	172.5 (2)	C33—C34—C35—C36	1.1 (4)
C46—C41—C42—C43	1.9 (3)	C34—C35—C36—C31	−1.7 (5)
N41—C41—C42—C43	−179.0 (2)	C32—C31—C36—C35	1.3 (4)
C41—C42—C43—C44	−1.4 (3)	N31—C31—C36—C35	−178.1 (2)

### Hydrogen-bond geometry (Å, º)

**Table:**

*D*—H···*A*	*D*—H	H···*A*	*D*···*A*	*D*—H···*A*
N1—H1*B*···O21	0.89	2.10	2.970 (2)	165
N1—H1*C*···O1^i^	0.89	1.86	2.743 (2)	172
O43—H43*A*···O2^ii^	0.82	1.81	2.623 (2)	171
O23—H23*A*···O32^ii^	0.82	2.08	2.820 (3)	150
O33—H33*A*···O2^iii^	0.82	1.88	2.687 (2)	170

Symmetry codes: (i) −*x*+1, *y*−1/2, −*z*; (ii) *x*−1, *y*−1, *z*; (iii) *x*, *y*−1, *z*.

### Geometric parameters of short ring-interactions with centroid to centroid distances less than 6.0Å.

**Table:**

	*Cg*–*Cg*(Å)	α(°)
*Cg*1···*Cg*4	4.7810 (14)	12.02 (12)
*Cg*1···*Cg*5	4.6231 (14)	14.12 (12)
*Cg*2···*Cg*4	4.3316 (15)	11.22 (13)
*Cg*2···*Cg*4^i^	5.9401 (15)	65.16 (13)
*Cg*2···*Cg*5	4.6336 (14)	14.58 (12)
*Cg*4···*Cg*5^ii^	4.3160 (14)	17.89 (12)
*Cg*5···*Cg*6^iii^	5.8571 (13)	61.42 (11)
*Cg*6···*Cg*6^iv^	3.5724 (12)	4.92 (2)

Notes: α is the dihedral angle between interacting planes; *Cg*1 is the centroid of the pyrrole ring; *Cg*2 is the centroid of the benzene indole ring; *Cg*4 is the centroid of C21 to C26 π-ring; *Cg*5 is the centroid of C31 to C36 π-ring; *Cg*6 is the centroid of C41 to C46 π-ring.Symmetry codes: (i) 1-x,-1/2+y,1-z; (ii) -1+x,y,z; (iii) 1+x,y,z; (iv) -x,1/2+y,-z

### Geometric parameters of N-O···*Cg*(π-ring) interactions with O..*Cg* less than 4.0Å.

**Table:**

	O···*Cg*(Å)	N–O···*Cg*(°)	N···*Cg*(Å)
N21–O21···*Cg*1	3.262 (2)	96.65 (15)	3.615 (2)
N21–O22···*Cg*2	3.821 (3)	74.05 (16)	3.678 (2)
N31–O31···*Cg*4^iii^	3.954 (2)	67.54 (15)	3.665 (2)

Notes: The centroids and symmetry codes are as in table A.
